# Case of Ununited Fracture of Both Bones of the Leg, Treated by Friction and the Starch Bandage

**Published:** 1842-11-26

**Authors:** 


In the Provincial Medical Journal, of Oct. 29th, a case of Ununited Fracture of both bones of the leg, successfully treated by friction and the starch bandage, is reported:

"The limb being firmly grasped with both hands, the fractured ends were made quickly, but not roughly, to rub upon each other, the action being continued for the space of two or three minutes. This produced a very considerable degree of pain, which was ill borne by the patient. The limb was then simply placed upon a pillow, and nothing more done till the following morning, when the same process of attrition was repeated, and continued on the two succeeding days. The ends of the bones were then adjusted, and encircled, herring-bone fashion, with strips of soap plaster, and an eighteen-tailed bandage applied, being previously well starched. Strips of linen, two or three times doubled, and about two inches in breadth, soaked in the same ingredient, were placed longitudinally on its outside and anterior aspect. The limb then received an additional support above and below by a wooden splint, the whole being ultimately secured with tapes. At the end of two or three days it became so completely encased, that no movement of the patient could in any degree disturb the adjusted ends of the bones. The apparatus was not removed until the end of six weeks, when, upon examination, it was found that union had taken place. The limb continued weak for a considerable time, and at the end of about ten weeks he was so far enabled to bear weight upon it, as to allow of his crutches being laid aside, and walked with the aid of two sticks."

